# How Fast is Famous Face Recognition?

**DOI:** 10.3389/fpsyg.2012.00454

**Published:** 2012-10-30

**Authors:** Gladys Barragan-Jason, Fanny Lachat, Emmanuel J. Barbeau

**Affiliations:** ^1^Centre de Recherche Cerveau et Cognition, Université de Toulouse, CNRS-UMR 5549Toulouse, France

**Keywords:** face detection, face recognition, gender categorization, familiarity, famous faces

## Abstract

The rapid recognition of familiar faces is crucial for social interactions. However the actual speed with which recognition can be achieved remains largely unknown as most studies have been carried out without any speed constraints. Different paradigms have been used, leading to conflicting results, and although many authors suggest that face recognition is fast, the speed of face recognition has not been directly compared to “fast” visual tasks. In this study, we sought to overcome these limitations. Subjects performed three tasks, a familiarity categorization task (famous faces among unknown faces), a superordinate categorization task (human faces among animal ones), and a gender categorization task. All tasks were performed under speed constraints. The results show that, despite the use of speed constraints, subjects were slow when they had to categorize famous faces: minimum reaction time was 467 ms, which is 180 ms more than during superordinate categorization and 160 ms more than in the gender condition. Our results are compatible with a hierarchy of face processing from the superordinate level to the familiarity level. The processes taking place between detection and recognition need to be investigated in detail.

## Introduction

**Figure 1:**
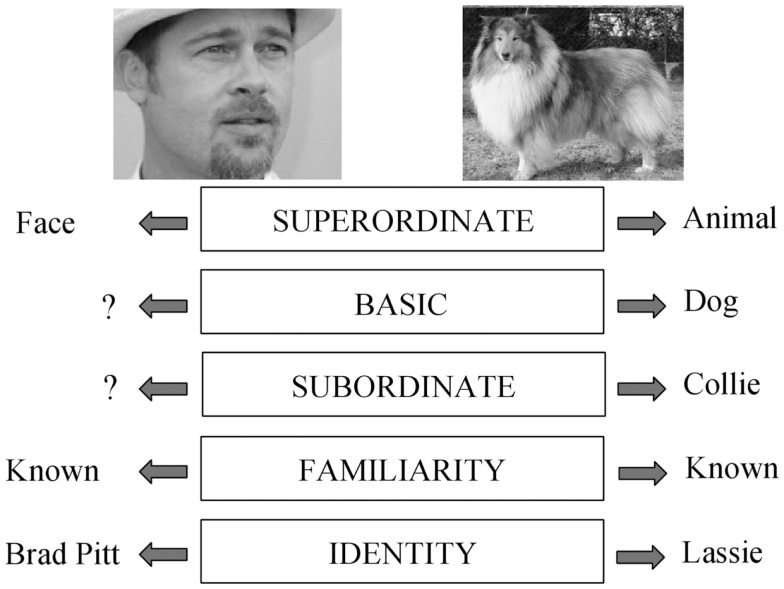
**Adaptation of Rosch’s model (Rosch, 1975)**. Different levels of processing. The picture on the right can be recognized (1) as an “animal,” (2) as a “dog” (basic level, which is usually the entry point), (3) as a “collie,” and even (4) as your own dog or a famous dog such as “Lassie.” The picture on the left can categorized (1) as a “(human) face” (superordinate level), (2) as known or not (familiarity level), or (3) as “Brad Pitt” (identity level).

Consider the picture of a face. It can potentially be processed at the superordinate level (this is a face, not an animal), at the familiarity level (I know this face), or at the identification level (this is the face of Brad Pitt). These different levels of processing, and related concepts, are presented in Figure 1, following an adaptation of Rosch’s hierarchical model of object recognition (Rosch, 1975). Most researchers in the field would probably agree that these different levels of processing are organized hierarchically. For example, most prosopagnosic patients have difficulty recognizing whether a face is familiar or not, but perform well on face detection tasks (e.g., Busigny et al., 2010). However, the reverse dissociation has not been reported. Such reasoning has led to the classic model of face processing proposed by Bruce and Young (1986), which in essence is a hierarchical, or sequential, model.

Comparisons of reaction times (RT) between superordinate and familiarity/identification levels support this idea of a hierarchy. It has been robustly shown that superordinate tasks (e.g., detecting human faces among other stimuli) can be performed quickly (Thorpe et al., 1996), with minimum RT around 250–290 ms (reviewed in Fabre-Thorpe, 2011). The minimum reaction time reflects the speed of the processes that are strictly necessary to make the first accurate behavioral responses (Rousselet et al., 2003). This 250–290 ms limit is now regarded as a reference when discussing the speed of the visual system and has been useful in attempts to identify the brain mechanisms underlying visual processes at the superordinate level (e.g., Liu et al., 2009; Mack and Palmeri, 2011; DiCarlo et al., 2012). In contrast, recognizing famous faces among unknown ones appears to take much longer (from 431 to 875 ms, Kampf et al., 2002; Herzmann et al., 2004; Caharel et al., 2005; Boehm and Paller, 2006; Paller et al., 2007; Baird and Burton, 2008). Even studies relying on priming, which should enhance speed, report rather lengthy RTs. For example, an influential study by Ellis et al. (1990) reported that recognizing famous faces yielded mean RTs of 862 ms when unprimed and 709 ms when primed, despite explicitly asking subjects to answer as fast as possible. Furthermore when subjects were required to answer before 600 ms, and only very famous faces were presented, mean RTs were still 598 ms when unprimed and 519 ms when primed. Although mean RTs were reported rather than minimum RTs, they still reflect much more time than when faces have to be processed at the superordinate level.

However, the evidence that faces are processed hierarchically from the superordinate to the identification level (Figure 1) is not that clear. The “entry point,” i.e., the “particular level of abstraction at which contact is first made with semantic memory” (Jolicoeur et al., 1984), is supposed to shift from the basic level of abstraction to the subordinate level (Figure 1) with increasing expertise (e.g., from “dog” to “poodle,” Rosch, 1975; Tanaka and Taylor, 1991; Tanaka and Curran, 2001). Experts develop efficient routines to recognize domain-specific objects and consequently humans, as face experts, could be expected to recognize faces very rapidly. In agreement with such a theory, some studies have reported that subjects are faster at identifying familiar faces at the level of the identity than they are at the superordinate level (Tanaka and Curran, 2001; Anaki and Bentin, 2009). In such tasks however, a category verification label procedure is used (e.g., “answer if the next picture is that of Brad Pitt”). Presenting such labels before the stimuli could allow subjects to rely on top-down processes (e.g., pre-activating features characteristic of Brad Pitt) that could speed up the recognition of stimuli for which such features are highly diagnostic. Therefore, such tasks are rather different from the tasks discussed above, where subjects recognized famous faces among unknown ones without any clue as to what was going to be presented. However, the possibility remains that humans, as face experts, process faces directly at the level of familiarity/identity, or else with only a marginal delay between the superordinate and the familiarity/identity level. In line with this idea, it is worth emphasizing that while superordinate tasks are usually run under speed constraints (i.e., subjects are asked to answer as fast as they can), this has usually not been the case for face recognition tasks. Therefore, the delay between superordinate and identity recognition tasks may be smaller than thought. In addition, the use of speed constraints appears critical in the present case since famous faces can, in principle, be recognized based on familiarity (known/unknown) or on identification (“this is Brad Pitt”), with identification probably taking more time than familiarity. Consequently, the time difference observed between the superordinate and the recognition tasks may occur because the subjects perform the recognition task at the identification level. This delay could be reduced if subjects were constrained to answer faster, based on familiarity.

In summary, there is some agreement on the speed at which faces can be categorized at the superordinate level, which provides a useful reference for time-limits since it imposes constraints on models of vision by limiting the number of plausible computational steps (Liu et al., 2009). We are not convinced that the speed with which famous faces can be recognized has been accurately measured, in particular using speed constraints. The main aim of the present study is to identify this speed. A secondary aim of the study is to assess the delay, if any, between the superordinate and familiarity/identity levels. The system allowing faces to be categorized at the superordinate level is indeed considered “fast” and its mechanisms are beginning to be understood. The comparison of face categorization at the superordinate and at the familiarity level could help to assess whether familiarity level categorization is “fast” or “slow” and whether it relies on mechanisms similar to those used in superordinate level categorization.

However, comparing superordinate and familiarity level categorization tasks raises a potential problem relating to diagnostic information. Diagnostic information is that deemed necessary and sufficient to perform a categorization task, and is defined by the interaction between task demand and stimulus availability (Schyns, 1998). Hence, task performance will not only vary according to task demands, but also according to the composition of the image set, including physical similarities between targets and non-targets. By definition, the exemplars within a superordinate category are less similar than the members of a subordinate category (Rosch, 1975), and so any difference found between the superordinate and familiarity levels could be related to the difference in the stimulus sets that were used. In order to overcome this problem, we therefore ran a control task in which subjects had to perform a gender categorization task using the same set of stimuli as in the familiarity task. All subjects therefore underwent three tasks (superordinate – familiarity – gender) under speed constraints.

## Materials and Methods

### Participants

Eighteen young subjects (10 females, median age: 24, range: 20–31 years, 1 left-handed), all Caucasian, with normal or corrected-to-normal vision participated in the main experiment. Seven other subjects (2 females, median age: 24, range: 21–31 years, all right-handed and Caucasian) performed a Control experiment. All subjects volunteered and gave their written informed consent to participate in the experiments.

### Stimuli

**Figure A1:**
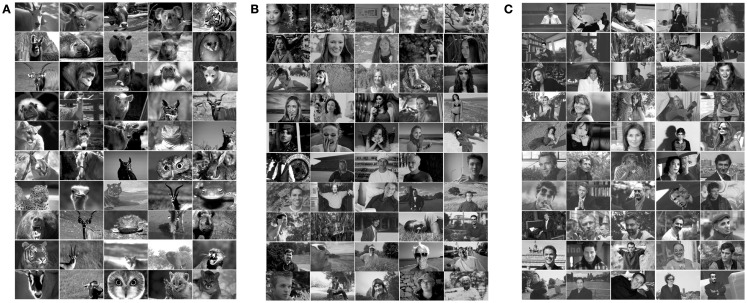
**Examples of animal (A), unknown (B), and famous (C) faces used in the Human/non-Human and the Famous/non-Famous categorization tasks**.

**Figure A2:**
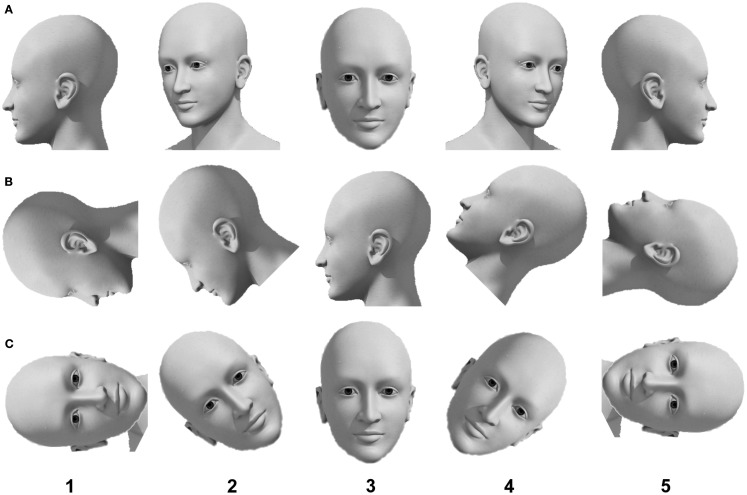
**Description of horizontal (A), vertical (B), and slope (C) orientations, with number 1 used when the face was orientated between the first and second position, 2 between position 2 and 3, 3 when the full-face was presented etc**. Details of the results are provided in Table A1.

Stimuli consisted of 360 grayscale photographs of animal faces from different subcategories (mammals, birds, reptiles…) used in a previous study (Rousselet et al., 2003), 540 photographs of non-famous human faces and 270 photographs of famous faces (Brad Pitt, Bill Gates…). Examples of the stimuli are presented in the supplementary methods (Figure A1 in Appendix). All faces were presented in their natural context (i.e., they were not cropped and some background could be seen) as natural scenes are processed efficiently by the visual system (Vinje and Gallant, 2000; Simoncelli and Olshausen, 2001). They were as varied as possible. Faces were presented at different scales and in different views. Photographs were randomly assigned to conditions across subjects (see below). We took care that the photographs of famous faces did not contain “obvious” specific information (e.g., like a Golden Globe statuette from the Hollywood Foreign Press Association). Photographs of unknown faces were chosen to look like those of famous people in terms of quality (professional photographs), attractiveness (most of the photographs were of models), and emotion so that subjects could not base their answers on these criteria. Each image was 320 × 480 pixels. In order to verify that the stimulus sets reached a satisfactory level of homogeneity, we performed statistics on the whole image including the background and we verified that the three sets had comparable mean gray-level values [luminance: *F*(2, 1167) = 0.489, *p* = 0.61] and standard deviation of gray-values [contrast: *F*(2, 1167) = 1.005, *p* = 0.37]. We also verified that animal faces, non-famous human faces, and famous faces did not differed in term of luminance [*F*(2, 1167) = 1.288, *p* = 0.28], contrast [*F*(2, 1167) = 1.667, *p* = 0.19], and size [number of pixels: *F*(2, 1167) = 0.927, *p* = 0.39] after having cropped them from the background manually. We controlled that male and female faces did not differ in size [*F*(1, 808) = 0.01, *p* = 0.94]. Furthermore, we also verified other variables such as head orientation, paraphernalia, race, and face expression between male and female faces on the one hand and famous and unknown faces on the other hand using *post hoc* analyses. Details are provided in Figure A2 and Table A1 in Appendix.

### Experimental setting

Subjects sat in a dimly lit room, 90 cm from a 19″ CRT computer screen (resolution: 1024 × 768; vertical refresh rate: 100 Hz) controlled by a PC computer. Stimuli subtended a visual angle of ∼7.2 × 10.7°. Photographs were displayed on a black background using Eprime^®^ software. Participants responded by raising their fingers from an infrared response pad (e.g., Rousselet et al., 2003) as quickly as possible.

### Experimental design

#### Main experiment

The experiment consisted of a go/no-go task divided into six blocks of 120 photographs (60 targets and 60 distractors). Two blocks were used in each condition (Human/non-Human; Male/Female or Female/Male; Famous/non-Famous). Importantly, half of the stimuli in the gender condition were famous and half non-famous faces chosen randomly from the same initial set of stimuli that were used in the Famous/non-Famous condition. Stimuli were randomly displayed within each condition. Famous faces were randomly distributed between Gender and Famous/non-Famous conditions across subjects. Unknown faces were randomly distributed across the three conditions across subjects.

Subjects were trained before each condition with a separate set of stimuli. These stimuli were used only in the training session in order to familiarize subjects with the go no-go task and the rapid presentation of the stimuli. These stimuli were never used again in any experiment. All subjects performed one training session except one subject who performed two. Subjects were instructed to raise their fingers from a response pad as quickly as possible when a target (human face in the Human/non-Human categorization task, famous face in the Famous/non-Famous categorization task) was presented (go response). Because some studies suggest that humans categorized same-sex targets more quickly (e.g., Zárate and Smith, 1990) and better (e.g., Wright and Sladden, 2003) than opposite-sex targets, subjects were instructed to answer as fast as possible when a male (for male subjects) or a female (for female subjects) was presented during the Gender categorization task. At the beginning of each trial, a fixation cross appeared for a random interval (300–600 ms), followed by a photograph flashed for 100 ms and a black screen for 900 ms. Stimuli were trial-unique in order to avoid repetition effect known to accelerate RTs (e.g., Ramon et al., 2011).

#### Control experiment

To determine whether reaction time in the Famous/non-Famous task was related to the ability to accurately identify the Famous faces, seven other subjects did another experiment in which they learned all famous faces. They first performed a Famous/non-Famous go/no-go task as described above [except that the task was divided into three blocks of 180 photographs (90 famous and 90 non-famous faces)].

Then, these subjects performed four additional sessions of the Famous/non-Famous task. They performed two additional sessions per week for 2 weeks. Each go/no-go session was preceded by a learning phase that consisted of learning all the target stimuli (the 270 famous faces), which remained the same over all five sessions. Subjects were explicitly instructed to learn all stimuli. Famous and unknown faces (which were different from non-famous face distractors used in the following Famous/non-Famous go/no-go task) were presented to the subject. Stimuli remained on the screen until subjects pressed one button if the face was famous, or another if the face was from the unknown set. After each response, feed-back appeared on the screen to enhance accuracy.

In summary, famous face targets were presented a total of nine times (four times during the explicit learning sessions, five times during the test sessions). Non-famous face distractors were not presented during the learning phase and were presented five times during test sessions.

### Speed constraints

Stimuli were flashed quickly (100 ms) and subjects had to answer within 1000 ms post-stimulus. Subjects performed training sessions with a separate set of stimuli before each task and could repeat the training if they wanted. Subjects were motivated to perform as quickly as possible by displaying median RT and false alarm rates after each block. Furthermore, they received strong encouragement before and between blocks to answer as fast as possible. In particular, after each block, they were asked to “beat” their RT score.

### Behavioral performance analysis

Performance (accuracy) was the percentage of correct go and no-go responses over the total number of stimuli. RTs under 200 ms (considered as anticipation, Rousselet et al., 2003; 2 go-responses out of 5848 analyzed in the present study) or above 1000 ms (38 responses) were discarded. RT latency analyses were performed over all data and across subjects (e.g., Rousselet et al., 2003). To obtain an estimation of the minimum processing time required to recognize targets, the latency at which correct go-responses started to significantly outnumber incorrect go-responses was determined. The minimum processing time required to recognize targets was calculated for the entire group as well as for each individual subject. At the group level, a Chi-square test on 10 ms time bins was used across trials (*p* < 0.001; Rousselet et al., 2003). In order to calculate the minimum processing time for each individual, a Fisher’s exact test on 30 ms time bins was used (*p* < 0.05). To correct for multiple comparisons, significance had to last 60 ms at least and the beginning of the first significant bin was considered as the minimum RT (Liu et al., 2009). A Kolmogorov–Smirnov test showed that minimum, median, and mean RTs calculated for each participant and for each image were not normally distributed. Therefore, a Wilcoxon test was applied to compare minimum, mean, and median RTs across conditions.

## Results

In the current experiment, we were interested in determining the minimum reaction time needed to either detect (Human/non-Human condition) or recognize (Famous/non-Famous condition) a face. Because these two conditions involved the use of different stimuli, subjects also performed a Gender categorization task on the same set of stimuli to those used in the Famous/Non-Famous condition.

### Main experiment

**Figure 2:**
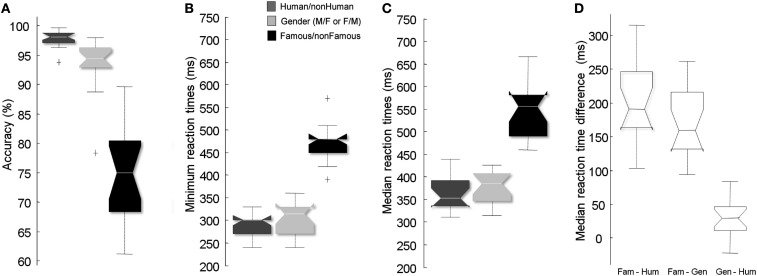
**Results across subjects in the three conditions**. Accuracy across subjects **(A)**, minimum RTs **(B)**, median RTs **(C)**, and intra-subject difference between median RTs **(D)** in the Famous/non-Famous and Human/non-Human conditions (left) and the Gender and Human/non-Human conditions (right). Positive score indicates that the participants are slower on the Famous/non-Famous task vs. Gender or Human/non-Human. “+” signs indicate outliers.

Figure 2A shows subjects’ performances. Subjects were more accurate in the Human/non-Human (97.8%, SD = 1.4) than in both Famous/non-Famous [74.9%, SD = 7.7; *Z*(17) = −5.11, *p* < 0.0001] or Gender [93.6%, SD = 4.5; *Z*(17) = 4.85, *p* < 0.001] conditions. Subjects were also more accurate in the Gender condition than in the Famous/non-Famous condition [*Z*(17) = −4.03, *p* < 0.001].

We compared RT values between conditions. Participants were slower in the Famous/non-Famous condition than they were in the Human/non-Human condition whether computing minimum [*Z*(13) = −5.17, *p* < 0.0001], mean [*Z*(17) = −5.11, *p* < 0.0001] or median [*Z*(17) = −5.11, *p* < 0.0001] RTs (Figures 2B,C). RTs in the Famous/non-Famous condition were also significantly slower than in the Gender condition [minimum: *Z*(13) = −5.14; *p* < 0.0001; mean: *Z*(17) = −5.11; *p* < 0.0001; median: *Z*(17) = −5.11; *p* < 0.0001]. RTs in the Gender condition were slower than in the Human/non-Human condition [minimum: *Z*(17) = −2.21; *p* < 0.02; mean: *Z*(17) = −2.99; *p* < 0.003; median: *Z*(17) = −2.20; *p* < 0.02]. The minimum RT in the Famous/non-Famous condition could not be calculated for four subjects because the ratio of correct/incorrect go-responses was not large enough.

In order to verify that the slow RTs reported in the Famous/non-Famous condition were not the result of a smaller number of responses (accuracy was lower in the Famous/non-Famous condition than in the two others), we conducted a bootstrap analysis by computing the surrogate distribution of the median, based on 3000 resampling of *n* randomly picked RTs (*n* corresponding to the number of trials obtained in the familiarity condition for each subject). We then computed the difference between conditions and estimated the 95% CI (confidence interval) of the distribution of mean difference across subjects. This analysis also showed a significant difference between the three conditions (*p* < 0.001) and confirmed that the significant time difference between the Famous/non-Famous task and the two others was not due to the smaller number of responses.

To determine if the time difference between conditions was observed in each subject, intra-subject differences between conditions were computed (Figure 2D). The average difference between the Famous/non-Famous and the Human/non-Human conditions across subjects was 182 (range: 120–300 ms) and 199 ms (range: 103–315 ms) for minimum and median RTs respectively. The average difference between the Famous/non-Famous and gender tasks was 158 (range: 120–240 ms) and 170 ms (range: 94–262 ms) for minimum and median RTs respectively.

We also performed a RT analysis across trials (Figure 4) in order to increase statistical power and again, a minimum difference of 190 ms was reported (250 vs. 440 ms) between the Human/non-Human and the Famous/non-Famous conditions and of 160 ms (280 vs. 440 ms) between the Famous/non-Famous and the Gender condition.

**Figure 3:**
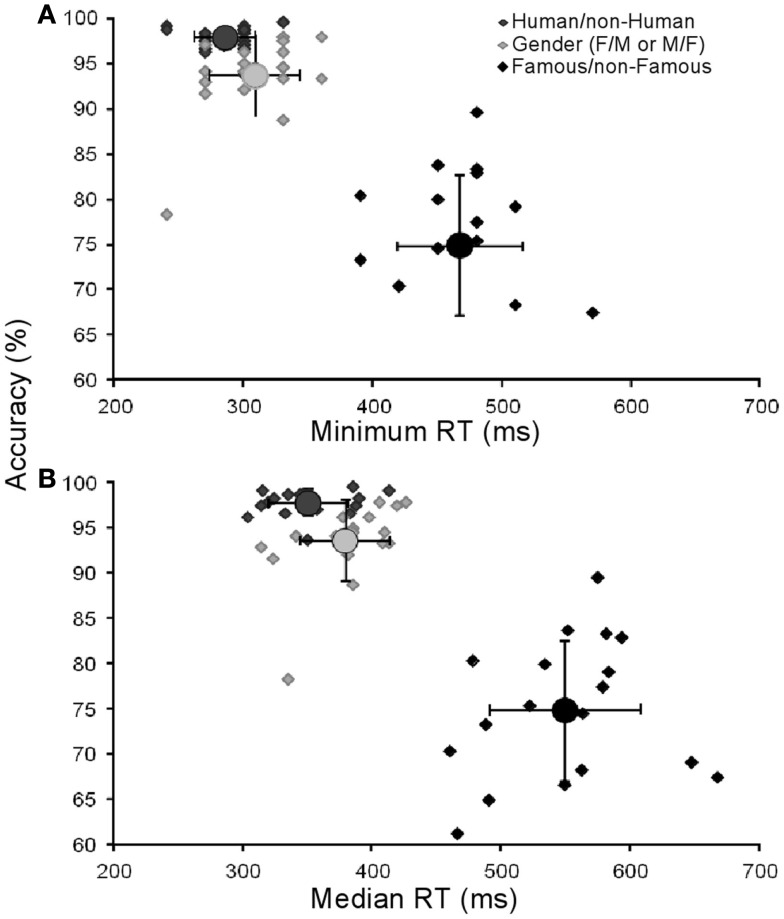
**Results for each participant**. Each point represents the performance of each subject (accuracy in functions of minimum in **(A)** and median reaction times in **(B)**).Circles represent the mean group performance. Vertical and horizontal lines represent SD. Minimum RTs could not be calculated for four subjects in the Famous/non-Famous task.

We investigated whether there was a relationship between famous faces RTs and accuracy, i.e., some faces could have been recognized faster with high accuracy and others slower with low accuracy. We computed the accuracy and median RTs for each famous face individually but no relationship was identified (*R*^2^ = 0.09). We also verified whether there was a relationship between accuracy and RTs across subjects. Again, no correlation was found in the Famous/non-Famous condition between accuracy and minimum or median RTs (*R*^2^ = 0.038/0.035 for minimum and median respectively). Individual results are presented in Figure 3.

To identify whether strategies differed across tasks, we determined the *C* index (bias index; Snodgrass and Corwin, 1988) and *d*′ for each subject in each condition. Details of these results are given in Table 1. Results showed that the *C* index was different in the Famous/non-Famous condition from each of the two other conditions [Famous/non-Famous vs. Human/non-Human: *Z*(17) = −4.83, *p* < 0.001; Famous/non-Famous vs. Gender: *Z*(17) = −4.41, *p* < 0.001], indicating that subjects were more conservative. *d*′ in the Famous/non-Famous condition was also significantly different from the two other conditions [Famous/non-Famous vs. Human/non-Human: *Z*(17) = −5.11, *p* < 0.001; Famous/non-Famous vs. Gender *Z*(17) = −4.63, *p* < 0.001].

**Table 1:** **Average results across participants**.

	Human/non-human	Famous/non-famous	Gender
**ACCURACY (%)**			
Mean	97.8 (1.4) [93.7–99.6]	74.9 (7.7) [61.2–89.6]	93.6 (4.1) [78.3–97.9]
Correct go	98.7 (1.9) [94.2–100]	58.1 (13.9) [34.2–81.71]	94.9 (3.6) [87.5–99.21]
Correct no-go	96.9 (2.1) [92.5–99.2]	91.7 (8.5) [73.3–99.2]	92.4 (7.4) [67.5–99.2]
*d*′	4.5 (0.60) [3.1–5.51]	1.8 (0.69) [0.78–2.9]	3.4 (0.67) [1.7–4.4]
*C*	−0.32 (0.37) [−0.82–0.41]	0.70 (0.35) [−0.04–1.23]	−0.06 (0.36) [−0.64–0.90]
**RT (ms)**			
Mean	359 (34) [310–439]	581 (66) [479–710]	397 (34) [336–446]
Median	350 (31) [303–413]	549 (58) [460–667]	379 (34) [314–426]
Minimum	285 (23) [240–3301]	467 (48) [390–5701]	308 (33) [240–3601]

*SD is indicated in round brackets. Range of individual responses (minimum and maximum) is indicated in square brackets*.

As some authors have argued that familiarity could have an effect on gender categorization (Rossion, 2002), we analyzed whether famous faces were categorized as male or female faster than unknown faces. We found no significant difference whether considering minimum [*Z*(16) = 0.054; *p* = 0.96], mean [*Z*(17) = 0; *p* = 1], median RTs [*Z*(17) = −0.16; *p* = 0.87], or accuracy [*Z*(17) = −1.57; *p* = 0.1]. The minimum RT of one subject could not be calculated because of too many false alarms.

### Control experiment

Participants were less accurate in the Famous/non-Famous condition (74.9%) than in both other conditions (>93%). We therefore tried to enhance accuracy in the Famous/non-Famous condition by having subjects learn all famous faces over five sessions (see Control Experiment in the Materials and Methods). Subjects’ accuracy rose from 72.8 to 89.6% [*Z*(6) = −3.08, *p* = 0.002; see Table 2 for details], which appears comparable to what subjects obtained in the main experiment during the Human/non-Human and the Gender conditions (Table 1). However, even if RTs across subjects decreased significantly (∼100 ms) from the first to the last session [minimum: *Z*(6) = 2.19, *p* = 0.02; median: *Z*(6) = 2.04, *p* = 0.04], RTs in the final session were still much slower (∼120 ms) than in the Human/non-Human condition.

**Table 2:** **Average results from control experiment**.

Session number	Minimum RT (ms)	Median RT (ms)	Accuracy (%)
1	500 (199) [390–600]	597 (218) [474–709]	72.8 (2.3) [68.3–92.4]
2	471 (159) [390–570]	570 (195) [468–639]	86.5 (5.3) [77.4–93.7]
3	437 (141) [360–510]	524 (175) [440–610]	88.8 (4.1) [83.1–94.6]
4	416 (135) [360–510]	497 (161) [442–557]	89.9 (4.8) [79.4–93.1]
5	402 (45) [360–450]	488 (54) [393–541]	89.6 (4.7) [83.9–95.6]

*Data from seven subjects except for session 5, which concerned only six subjects. SD is indicated in round brackets. Range of individual responses (minimum and maximum) is indicated in square brackets*.

## Discussion

**Figure 4:**
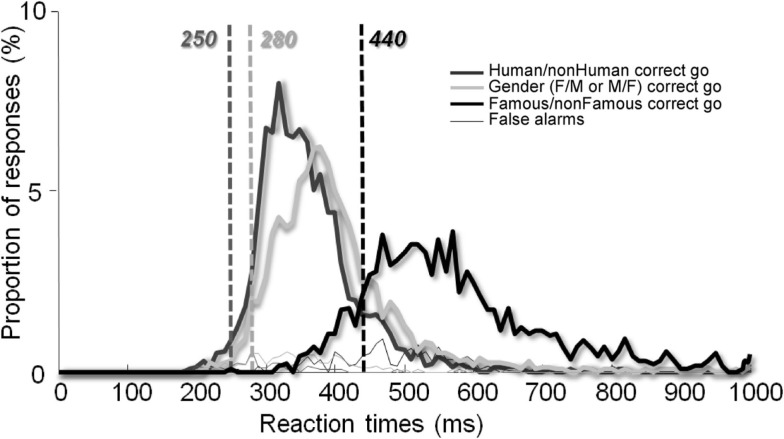
**Reaction time distribution across trials**. The vertical lines show minimum RTs for each condition.

To some extent, our Human/non-Human categorization task can be compared to the case of detecting the presence of a human in a scene (e.g., the face of a hiker in the mountains). On the other hand, our Famous/non-Famous task can be compared with the ecological situation of walking in the street and unexpectedly bumping into an acquaintance. In this study, we show that subjects are, in general, slow when they have to recognize faces, even under speed constraints. Their minimum RT was 467 vs. 285 ms in the Human/non-Human categorization task. Analyses of median RTs between the two conditions revealed a similar difference. Even when data were analyzed across trials to increase statistical power, a 190 ms time cost was observed between the two conditions (see Figure 4). In other words, once a face has been detected, processes lasting around ∼190 ms are needed to decide whether it is familiar or not. This is a huge (50%) increase.

The rationale for choosing the Human/non-Human categorization task as a reference was that it is widely agreed that such a task can be performed “fast.” Furthermore, different theories have been proposed to account for the efficiency with which humans categorize objects (Fabre-Thorpe, 2011). However, this raises an issue since both the tasks and stimuli were modified between conditions (Schyns, 1998), although the tasks were based on exactly the same paradigm. Furthermore, stimuli were obviously more similar in the Famous/non-Famous task (all were human faces) than in the Human/non-Human categorization task (human faces vs. animal faces). The time cost that we report between the two conditions can therefore, in principle, come either from the task or from visual similarity in the Famous/non-Famous condition.

This issue was addressed in the Gender categorization task. To the best of our knowledge, it is the first time that a gender task has been run under speed constraints and for which minimum RT are provided. Stimuli were the same as those in the Famous/non-Famous condition (unknown and famous human faces intermixed), and the paradigm was also the same (fast go/no-go on either males or females). The fast gender categorization task was performed with a minimum RT of 280 ms across trials (308 ms across participants). This is much less than the 440 ms (or 467 across participants) that we found in the Famous/non-Famous condition, even for the fastest subjects. In contrary to previous work (Rossion, 2002), no effect of famousness on gender categorization was observed. These results support the idea that the time cost observed between the two conditions in our study was not simply due to visual similarity in the Famous/non-Famous condition. As mentioned previously, the set of stimuli and the paradigm were the same in both the Famous/non-Famous and Gender conditions. RTs differed largely however, suggesting that subjects did not perform the same task. It is possible that subjects were fast in the Gender condition because they could base their answer on simple features (haircut for example), i.e., the task was a feature detection task. In contrast, this implies that face familiarity, when subjects have no clue about which person is going to be presented next, cannot rely on such simple visual mechanisms.

A second issue is related to the fact that accuracies in the Human/non-Human and gender conditions were significantly higher than in the Famous/non-Famous condition. This suggests that task difficulty could account for the time delay observed between conditions. A difference in accuracy was however expected. Since we used famous faces, we were also simultaneously probing each subject’s cultural knowledge, which varies greatly in a population. There are some arguments against the idea that task difficulty alone can account for the delay that was observed. First, we performed a correlation analysis between accuracy and mean RT computed for each famous face and no correlation was found. This result indicates that famous faces that were easily recognized by most subjects were not recognized faster. Second, subjects who performed well on the familiarity condition (e.g., accuracy above 1 SD, see Figure 3) did not appear to perform either slower or faster than the rest of the group. In accordance, no correlation between accuracy and median or minimum RTs was found across subjects. In a control experiment, subjects were familiarized with all famous faces on five occasions before being tested. Their accuracy rose to ∼90% but their minimum RT was still 402 ms (median: 488 ms, see Table 2), that is about 120 ms more than in the Human/non-Human condition. Overall, this argues in favor of the idea that the time cost observed between the Famous/non-Famous condition and the two other conditions is not simply due to task difficulty but to the fact that subjects, despite the paradigm being the same, performed the task differently.

The analysis of the bias indicated that subjects were significantly more conservative when they categorized faces as familiar than when they performed the Human/non-Human or gender categorization tasks (see Table 1). In our view, this result is in agreement with the idea that subjects perform a different task and could be explained by the fact that subjects access a person-related knowledge system during the Famous/non-Famous condition. This person-related knowledge system is strongly related to the social cognition system (both share largely overlapping neural substrate, e.g., Olson et al., 2007; Zahn et al., 2009) and would change the subjects’ bias (i.e., subject want to minimize false alarm, e.g., the probability to wrongly recognize a person).

Categorization at the Human/non-Human level has been suggested to rely on the first feed-forward sweep of activity through occipito-temporal areas (Thorpe et al., 1996). Recently, it has been proposed that rapid categorization could rely on the magnocellular pathway, i.e., on coarse but fast representations of objects that could guide slower parvocellular processing (Fabre-Thorpe et al., 2001; Macé et al., 2009; Fabre-Thorpe, 2011). Here, we find that recognizing faces takes around 190 ms in addition. What happens during this additional time?

Three main hypotheses can be formulated. First, the ability to rapidly recognize familiar faces could rely on the same feed-forward mechanisms that have been posited for superordinate categorization. However, more areas would need to be recruited in the so-called face-area network, hence taking more time. In a recent review, DiCarlo et al. (2012) suggested that the ventral stream can achieve fast feed-forward object recognition through a cascade of local circuits basically applying the same processing rule. These local circuits would progressively achieve image invariance through a cascade of “identity-preserving image transformations.” Although this model was mainly intended to explain object recognition at the superordinate or basic level, it can also be extrapolated to recognition at the individual level. In this case, more time would be needed simply because more local circuits (down the ventral stream) would need to be recruited. Whereas superordinate categorization probably involves posterior areas along the visual ventral stream, face recognition involves areas up to the most anterior part of the visual ventral stream (i.e., the temporal poles). Convergent evidence implicates the anterior temporal lobes in person processing, including studies of brain-lesioned patients showing person agnosia (Joubert et al., 2004, 2006), fMRI studies (Haxby et al., 2000), or single neuron recordings in macaque monkeys (Freiwald and Tsao, 2010). It is also in these areas that “person-specific” neurons of the sort needed to identify famous persons are found (Quiroga et al., 2005).

An alternative has been put forward by Hochstein and Ahissar (2002), who distinguish vision-at-a-glance, based on implicit and automatic processes along the feed-forward hierarchy, from vision-with-scrutiny, based on conscious processes beginning at the top of the hierarchy and “gradually returning as needed” to the ventral stream (Hochstein and Ahissar, 2002; Hegdé, 2008) in order to refine perceptual processes (Bullier et al., 2001). Goffaux et al. (2011) found that face-selective anterior infero-temporal regions responded to brief presentation of faces filtered at low spatial frequencies, suggesting that these areas could indeed receive a first-pass of coarse information. Likewise, using intracerebral recordings in epileptic patients, we found evidence of an early period of activity (∼130 ms post-stimulus) in the inferior frontal gyrus during a famous face recognition task. However, it was followed by a period of massive parallel processes in the whole visual ventral stream at 240 ms, during which vision-with-scrutiny could take place (Barbeau et al., 2008). Interestingly, Sugase et al. (1999) recorded neurons from the macaque temporal cortex and found that category information was conveyed in the first part of the neuronal firing rate, while fine information about identity was conveyed at a slower rate with a delay of about 50 ms. They hypothesized that this time delay could allow inter-area and feed-back communication. Within this framework, the delay we identified from the Human/non-Human to Famous/non-Famous condition could be related to such processes, which are specifically needed to refine visual information when faces have to be recognized. In this case, face recognition would not rely on feed-forward mechanisms alone. Face recognition could to some extent be considered as a type of categorization (known/unknown) at a subordinate level, as suggested by the time cost observed between the Human/non-Human and Famous/non-Famous conditions. However, it is not any kind of subordinate level. It is the most refined level of subordinate processing, where stimuli are processed at the specific and single exemplar level. It could therefore be discussed whether subjects at this level perform a “category” since there is only one exemplar of its kind. In our view, subjects clearly go beyond categorization to focus on the exemplar level (i.e., try to retrieve as much information as possible about the face they attempt to recognize). Furthermore, face recognition has the particularity to be accompanied by a sense of familiarity when faces are known (e.g., the butcher-in-the-bus phenomenon, which corresponds to the situation of recognizing someone as familiar, while at the same time being unable to remember the circumstances of any previous meeting or anything else about the person, e.g., Yovel and Paller, 2004). Because of these particularities, face recognition probably relies on more complex visual mechanisms than suggested in the feed-forward model, implying that it also relies on different mechanisms than those used for superordinate processing.

The third possibility is that subjects activate additional neural systems, notably those related to person-knowledge, on top of, and after, the visual processes. These systems are thought to rely on the temporal poles (Gainotti et al., 2008) and could be accessed only after familiarity with the face has been assessed.

These three possibilities do not contradict each other. Two subjects were able to answer very fast in the Famous/non-Famous condition at ∼390 ms (outliers in Figure 2B, see also Figure 3). We suggest that these fast subjects can perform based on familiarity only and that their performance may rely on a first-pass through the whole visual ventral stream up to the highest areas in the perirhinal cortex. However, most subjects cannot refrain from accessing the person-knowledge system before answering (as suggested by a different bias level). For example, Bruce and Young (1986) suggested that trying to identify a person was compulsory since we are “generally not satisfied that we know a face until more than a sense of familiarity is achieved.” Therefore in these subjects, both a period of feed-back processing to refine visual information *and* simultaneous access to person-knowledge stores would be necessary before they can answer, which could account for the 190 ms time cost between the conditions we found in most subjects. The 100–190 ms delay found in this study imposes constraints on neural models of vision, not by limiting the number of plausible computational steps, but rather because models have to account for such lengthy delays.

In conclusion, we show that subjects are relatively slow at recognizing famous faces despite the use of speed constraints. In particular, they are slower than when they have to process faces in Human/non-Human or Gender categorization tasks. These results may appear to be at odds with previous studies that have contrasted face processing at the superordinate and identity levels (Tanaka, 2001; Anaki and Bentin, 2009), which found exactly the opposite pattern of results: faster RTs at the identity level than at the superordinate level. However, a category verification label procedure was used in these tasks which could have resulted in “top-down” recognition. Our task relied on “bottom-up” recognition: we used a large pool of faces, thus preventing subjects from pre-setting the visual system for some specific diagnostic cues. It could be that the visual hierarchy is always respected in bottom-up (purely visual) recognition (Macé et al., 2009), but that it can be disrupted when verbal/visual paradigms are used.

## Conflict of Interest Statement

The authors declare that the research was conducted in the absence of any commercial or financial relationships that could be construed as a potential conflict of interest.

## Appendix

**Table A1:** **Comparison of visual characteristics across groups of photographs: head orientation (horizontal, vertical, slope, see Figure A2); race; other [paraphernalia, teeth being visible or not, presence of an expression (here limited to the presence of a smile or not since it was the only expression shown)]**.

Rate/category	Male	Female	*p*	Famous	Non-famous(%)	*p*
**HORIZONTAL**						
1	1.0	5.0	0.007*	2.9	3.1	0.1
2	15.0	16.7	0.84	17.1	16.1	0.61
3	61.7	59.7	0.35	56.7	62.2	0.67
4	18.3	17.0	0.47	21.3	15.3	0.052
5	4.0	1.7	0.067	2.1	3.3	0.42
**VERTICAL**						
1	0.3	1.0	0.35	0.8	0.6	0.65
2	7.7	4.7	0.093	3.8	8.1	0.054
3	88.7	90.3	0.59	90.4	88.9	0.47
4	3.3	4.0	0.78	5.0	2.5	0.089
5	0.0	0.0	No > value	0.0	0.0	No > value
**SLOPE**						
1	0.7	1.0	0.71	0.4	1.1	0.38
2	7.3	10.0	0.38	2.9	11.4	0.0006*
3	85.3	80.7	0.18	88.3	81.1	0.16
4	6.7	7.7	0.81	8.3	5.8	0.19
5	0.0	0.7	0.17	0.0	0.6	0.26
**RACE**						
Caucasian	92.7	91.7	0.37	94.2	92.5	0.46
Black	5.3	4.0	0.35	5.4	3.3	0.18
Asian	2.0	4.3	014	0.4	4.2	0.007*
**OTHER**						
Paraphernalia	29.3	36.3	0.30	34.6	31.7	0.35
Smile	48.7	59.3	0.23	49.2	57.2	0.36
Teeth	37.3	50.0	0.068	40.8	45.6	0.62

*A chi-square test was applied (between male and female face on the one hand and famous vs. non-famous face on the other hand) for statistical comparisons. Values are reported in proportion (%) and the * sign corresponds to a significant *p* value. The *p*s here were uncorrected for multiple comparisons. Three comparisons were found to be significant. Please note however that they are related to small percentage of photographs*.
